# Genetic testing for maturity-onset diabetes of the young resulting in an upgraded genetic classification of an HNF1A gene variant: a case report

**DOI:** 10.3389/fendo.2023.1173471

**Published:** 2023-06-16

**Authors:** Naama Pollack-Schreiber, Benjamin Udoka Nwosu, Parissa Salemi

**Affiliations:** Division of Pediatric Endocrinology, Cohen Children’s Medical Center of New York, New Hyde Park, NY, United States

**Keywords:** case report, MODY, HNF1A, diabetes mellitus, mutation

## Abstract

The frequent misdiagnosis of MODY (Maturity-Onset Diabetes of the Young) subtypes makes it necessary to clarify the clinical spectrum of the disease phenotypes in suspected subjects so that accurate diagnosis and management plans can be introduced as early as possible in the course of the disease. We report the case of a MODY subtype that was initially characterized as variant of uncertain significance (VUS) but was later changed to a likely pathogenic variant following our report of two cases where the full expression of the clinical phenotype was described. HNF1A-MODY (Maturity Onset Diabetes of the Young type 3) is one of the most common subtypes of MODY. Due to its variable clinical presentation, and the concerns with being misdiagnosed as either type 1 or type 2 diabetes, DNA sequencing is needed to confirm the diagnosis. This case report illustrates the clinical scenario leading to the identification of the gene variant c.416T>C(p. Leu139Pro) in the HNF1A gene, initially reported as a VUS and later upgraded to a likely pathogenic variant. Though the mutation was described in two Czech family members in 2020, the clinical course and phenotype was not characterized. Therefore, there was the need to fully describe the spectrum of the disease arising from the mutation. The case report fully describes the clinical spectrum of this mutation and provides much needed clinical management approaches to the wider scientific community.

## Introduction

Diabetes mellitus is characterized by hyperglycemia resulting from defects in insulin synthesis, secretion, and action. Sustained hyperglycemia leads to long-term complications such as kidney disease, ocular disease, and neuropathy (1). Diabetes mellitus is a group of metabolic diseases which was classified by the American Diabetes Association into 4 major forms in 1997. These forms include type 1 diabetes resulting from an autoimmune-induced destruction of pancreatic β-cells, and type 2 diabetes resulting from a combination of increased β-cell apoptosis and insulin resistance. A third category includes other specific types of pathologies such as monogenic diabetes syndromes (maturity-onset diabetes of the young (MODY), neonatal diabetes), diseases of the exocrine pancreas (pancreatitis, cystic fibrosis), and chemical/drug induced diabetes (like high-dose glucocorticoid therapy). The fourth form is gestational diabetes mellitus that is first diagnosed during pregnancy (2).

MODY is a form of monogenic diabetes arising from a collection of genetically inherited, non-autoimmune disorders that are present in childhood. Thirteen MODY genes have been characterized to date (3). The prevalence of monogenic diabetes is 1/10,000 in adults and 1/23,000 in children, though this could represent an underestimation because of the high frequency of misdiagnosis of MODY as either Type 1 or Type 2 diabetes, and the exclusion of non-White subjects in the population studies (3).

There are no clinical markers for MODY, therefore DNA sequencing is used to confirm the diagnosis when clinical suspicion exists. Shields et al. (2012) developed clinical prediction models that calculate an individual’s probability of having MODY compared with type 1 or type 2 diabetes. Those models showed improvement in the specificity (94% vs 91%) and sensitivity (91% vs 72%) in identifying MODY compared with the standard criteria (patient diagnosed before the age of 25 years and has an affected parent) (4). The use of Next-Generation Sequencing (NGS) methods has led to the confirmation of cases and the discovery of multiple novel MODY gene variants (5).

This case report presents the clinical scenario that led to the identification of a heterozygous mutation in the HNF1A gene which was initially reported as a variant of uncertain significance but was later upgraded to a likely pathogenic variant based on our two reported cases. This variant was previously identified in a patient in Czech Republic, that was included in a functional analysis of HNF1A-MODY variants (6). However, this is the first case in the United States that fully characterizes the phenotype and course of the diabetes associated with this mutation. Our report indicates that this is a pathogenic mutation leading to MODY as detailed in this case presentation.

## Case presentation

The patient is a 12-year-old boy of Indian ethnicity with no significant past medical history except for premature birth at 32 weeks of gestation. Routine laboratory studies obtained at his annual well-child physical examination at age 10 years and 6 months had revealed the following: non-fasting blood glucose of 158 mg/dL, glucosuria, and an elevated hemoglobin HbA1c of 8.4% (normal range 4.4-5.6%). His repeat laboratory studies at our Endocrinology clinic showed an HbA1c of 8.7% and a non-fasting serum glucose level of 189 mg/dL.

His history at the Endocrinology clinic was remarkable for an increased appetite, but negative for polyuria, nocturia, weight loss, hearing impairment or loss of color vision. His family history was remarkable for diabetes mellitus that spanned more than 3 generations. Affected individuals include the patient’s mother, maternal grandmother, maternal aunt, and maternal great grandfather.

His physical examination revealed that he was at the 98^th^ percentile for height, and at the 75^th^ percentile for BMI. He had no acanthosis nigricans and the rest of his examination was normal.

His laboratory investigation was negative for diabetes associated autoantibodies, namely glutamic acid decarboxylase antibodies, anti-insulin antibodies, anti-islet cell antibodies, and zinc transporter 8 antibodies. Following his initial evaluation, he was started on a basal bolus insulin regimen, with total daily dose of 0.22 units/kg/day. Over the subsequent 4 months, his long-acting insulin was gradually weaned and was discontinued when his HbA1c decreased to 6.1%. He was continued on only 2-2.5 units of premeal short-acting insulin daily (0.06 unit/kg/day). His diabetes associated autoantibodies remained negative upon retesting.

**Figure 1:**
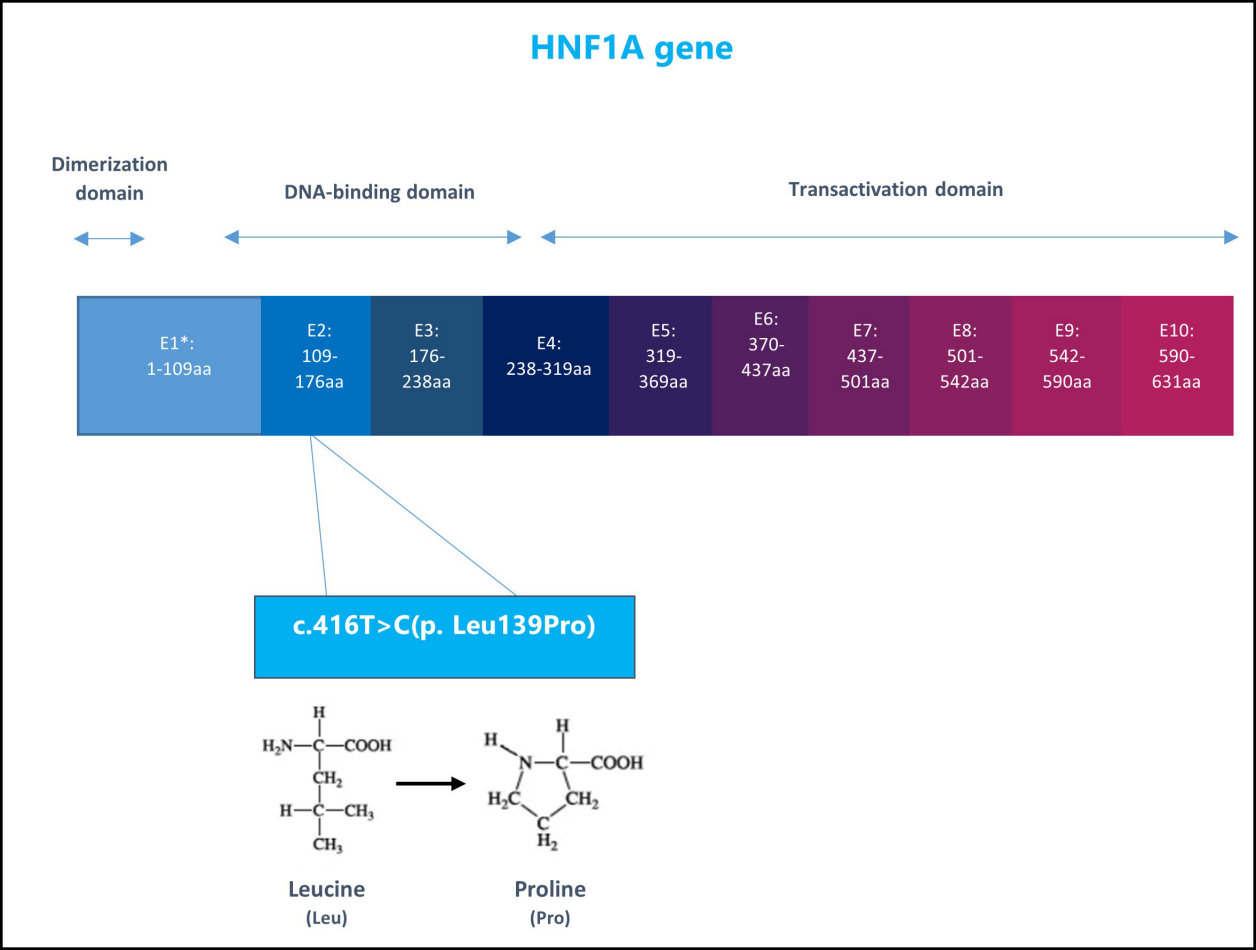
The structure of HNF1A gene and the patient specific genetic variant: nucleotide 416 in the coding DNA changed from Thymine (T) to Cytosine (C) leading to amino acid change in position 139 in the protein sequence, from Leucine to Proline. E1, Exon1.

He was referred to the Genetics clinic for evaluation for possible monogenic diabetes. His Invitae Monogenic Diabetes Panel showed a heterozygous mutation in the HNF1A gene, exon 2, variant c.416T>C (p. Leu139Pro), **Figure 1**. The sequence change replaces leucine, with proline, at codon 139 of the HNF1A protein. At the time of testing, in April 2022, this variant was not present in the Genome Aggregation Database (gnomAD) so it was initially considered a variant of uncertain significance.

Following the identification of our patient’s gene variant, the same variant was subsequently identified in his 42-year-old mother who is of normal weight and had received a diagnosis of type 2 diabetes mellitus at 31 years of age. This was shortly after the patient was born and when his mother’s HbA1c was 8.6%. She was placed on metformin and has undertaken lifestyle modifications. Following the diagnosis, we recommended to consult with her endocrinologist and consider changing the treatment to a sulfonylurea. Her most recent HbA1c in August of 2022 was 7.2%. The maternal grandmother and maternal great-grandfather have also been diagnosed with diabetes but reside in India and therefore have not undergone genetic testing. The additional identification of this mutation in the patient’s mother led to the upgrade of the classification from a “variant of uncertain significance” to a “likely pathogenic” variant.

**Figure 2:**
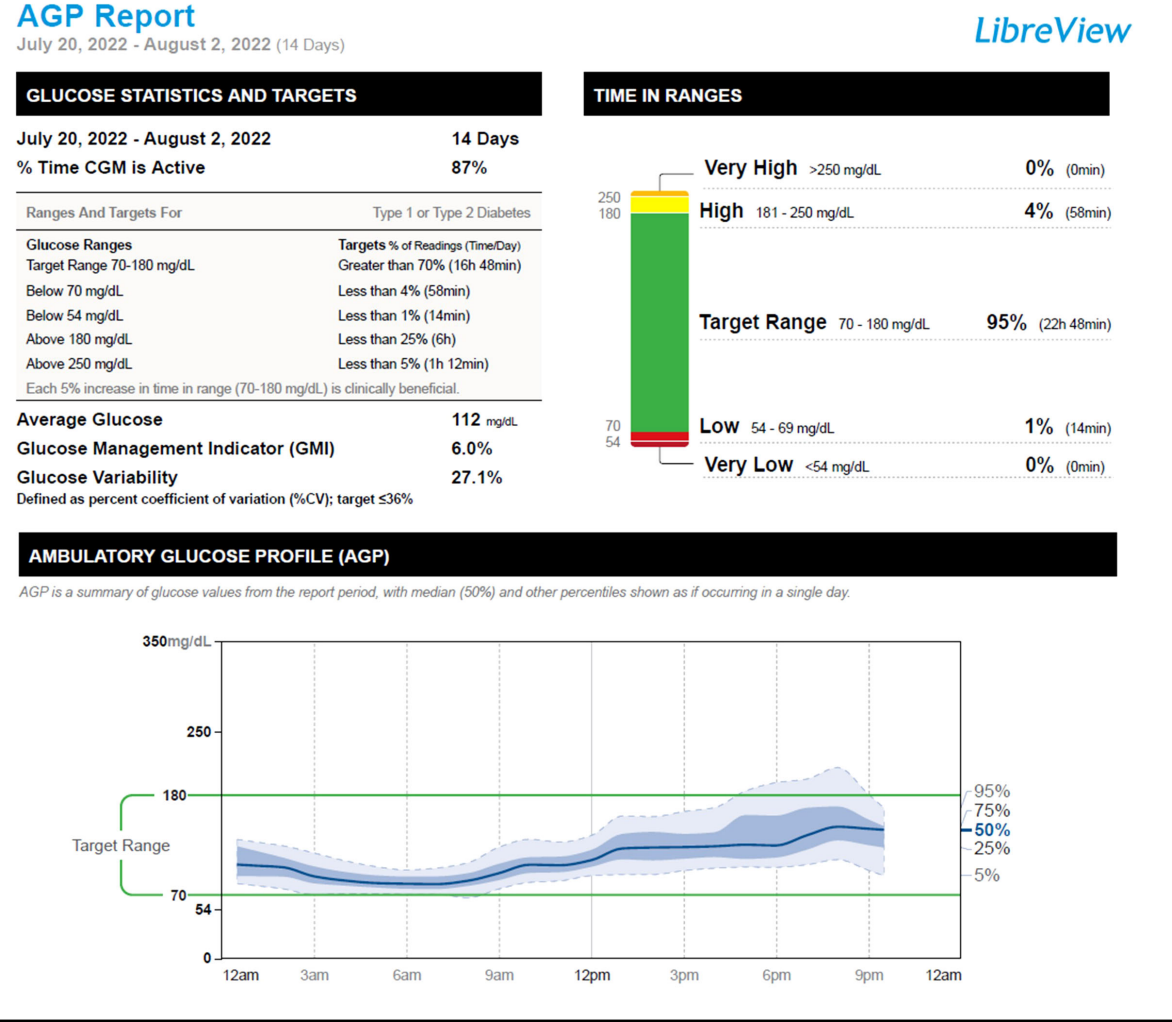
The patient’s CGM (Libre 2) report in the 2 weeks following insulin discontinuation.

**Figure 3:**
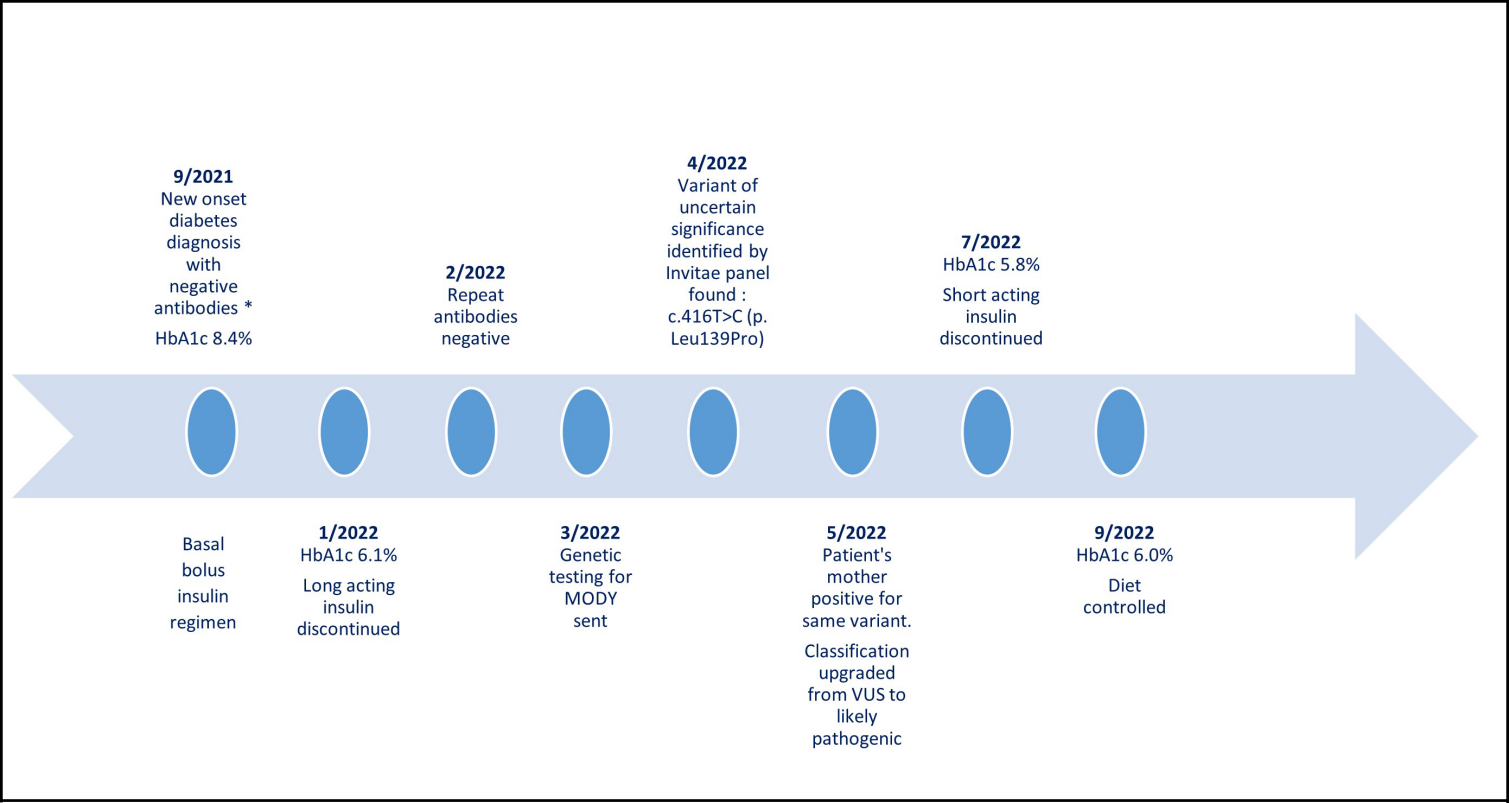
The patient’s timeline from diabetes mellitus diagnosis until today. *Diabetes antibodies: glutamic acid decarboxylase antibodies, anti-insulin antibodies, ant-islet cell antibodies, zinc transporter 8 antibodies. VUS, variant of uncertain significance.

Given the results of his molecular studies, in addition to his excellent glycemia and HbA1c level of 5.8%, his short-acting insulin therapy was discontinued. We monitored his glycemia using a continuous glucose monitor (CGM). Two weeks after the discontinuation of his insulin therapy, his CGM metrics showed a time in range (70-180 mg/dL) of 95%, time above range (181-250 mg/dL) of 4%, and time below range (54-69mg/dL) of 1%, **Figure 2**. One month following the discontinuation of his insulin therapy, his fasting and premeal glycemia were in the acceptable range: fasting glucose range of 82-113 mg/dL, and pre-meal glucose levels of 95-150 mg/dL. He has remained off insulin therapy for 7 months and maintains his glycemia on diet alone. **Figure 3**.

## Discussion

The frequent misdiagnosis of MODY subtypes makes it necessary to clarify the clinical spectrum of phenotypes in these subjects so that accurate diagnosis and management plans can be introduced very early in the course of the disease. Though the mutation was described in two Czech family members in 2020, the clinical course and phenotype was not characterized. Therefore, there was the need to fully describe the spectrum of the disease arising from the mutation. This case report highlights the clinical features seen in an individual with a medical course that led us to explore a diagnosis of monogenic diabetes.

HNF1A-MODY (MODY 3) is a monogenic disease with autosomal dominant inheritance due to HNF1A (hepatocyte nuclear factor 1 alpha) haplo-insufficiency caused by mutations in the *HNF1A* gene which codes the transcription factor HNF1α. This is one of the most common subtypes of MODY. HNF1A is located on chromosome 12, in the region 12q24.2. An extensive publication summarizing *HNF1A* variants recognizes 414 mutations in the HNF1A gene found in 1,247 families (7). The Exome Aggregation Consortium (ExAC) database presents 894 *HNF1A* variants, while the Genome Aggregation Database (GnomAD) presents 1231 variants spanning from the *HNF1A* promoter to 3´UTR region (8). More mutations were found in *HNF1A* exon 2 and exon 4. Patients with *HNF1A* mutations in exons 8-10 usually present with later-onset MODY 3 compared to patients with mutations in exons 1-6 (5).

In a study by Malikova et al. (2020), functional analysis was done on 17 *HNF1A* variants which were identified in 48 individuals from 20 Czech families presenting with diabetes. Among the 17 identified variants, 12 had not been previously reported in the literature, one of them being the c.416T>C (p. Leu139Pro) that was found in our patient and in two individuals from a single Czech family for whom a phenotype was not completely described. The variants were classified using the American College of Medical Genetics and Genomics (ACMG) guidelines into one of the following groups: pathogenic, likely pathogenic, uncertain significance, likely benign, and benign. In the study, the researchers used bioinformatics *in silico* tools and functional protein analyses (protein expression, transactivation, nuclear localization, and DNA binding). The variant p.Leu139Pro demonstrated only 8% of the transcriptional activity, severely reduced DNA-binding ability (13%), and reduced nuclear level activity (54%) (6).

The genetically confirmed mutation in our patient and his mother combined with the report of the deleterious effect of this variant *via* functional analysis led the widely used medical genetic testing company, Invitae, to upgrade the mutation classification to a likely pathogenic variant.

Classification of the genetic basis of hyperglycemia has implications for treatment. In cases of HNF1A-MODY, treatment depends on HbA1c levels and the age of the patient. If HbA1c is lower than 6.5%, a low carbohydrate diet may be temporarily successful. With a higher or rising HbA1c, the next useful treatment is sulfonylurea derivatives (5). Sulfonylurea derivatives increase endogenous insulin secretion, which allows the body to respond spontaneously to glycemic changes. In a study by Shepherd et al. (2009), 79% of patients diagnosed with HNF1A MODY and who were treated with insulin at the time of genetic testing, transitioned to treatment with sulfonylureas. 71% remained off insulin. Most patients experienced no deterioration in their glycemic control at a median of 39 months. Patients who required insulin treatment were diagnosed with diabetes for a longer period of time. This highlights the importance of an early genetic diagnosis and early initiation of sulfonylurea treatment (9). The sensitivity of patients with HNF1A-MODY to sulfonylurea treatment is due to reduced hepatic degradation which results in higher levels and longer persistence of sulfonylurea in the plasma; therefore, small doses of short-acting sulfonylureas are sufficient (10). In the presented case, a small dose of a sulfonylurea would be considered if persistent hyperglycemia reoccurs.

Proper recognition and diagnosis of MODY can significantly improve a patient’s quality of life by altering treatment from injections to either oral hypoglycemic agents or lifestyle modification alone. Early diagnosis may decrease the need for using insulin treatment. Clinical prediction models (4) are easy to use and may help determine which patients would benefit from molecular genetic testing. Reporting new mutations and their phenotypes is important in classifying the various mutations leading to MODY. Routine genetic testing in suspected individuals will add to the current knowledge.

## Data availability statement

The original contributions presented in the study are included in the article/supplementary material. Further inquiries can be directed to the corresponding author.

## Ethics statement

Written informed consent was obtained from the participant/patient(s)' legal guardian/next of kin for the publication of this case report.

## Author contributions

NP-S is the first author who completed the literature review and wrote the manuscript together with PS, the senior author. BN reviewed, edited and provided critical feedback to the manuscript final version. All authors contributed to the article and approved the submitted version. 
